# Supplementary material for: Complexome profiling of the Chlamydomonas psb28 mutant reveals TEF5 as an early PSII assembly factor
Source: Plant Cell. 2025 Mar 17;37(6):koaf055. doi: 10.1093/plcell/koaf055 (PMC12164590; doi:10.1093/plcell/koaf055)
Supplement: koaf055_Supplementary_Data [file koaf055_supplementary_data.zip › psb28_interactive.zip!/plots/intensity/qualityPlots/peptides/_Cre17.g706850.t1.1.html]

### Cre17.g706850.t1.1

Trivial name:   
SeqCoverage [median]: 7.60  
Mass: 34.60 kDa  
  
 

| Sequence | MissedCleavage | Mass [Da] | wtIntensity | muIntensity | Score |
| --- | --- | --- | --- | --- | --- |
| AAQYTLPVDSADANR | 0 | 1590.76 | 0.00 | 1373.60 | 96.64 |
| LVEPVYGSK | 0 | 990.54 | 0.00 | 450.44 | 61.96 |
|  |  |  | 0.0 | 1824.0 |  |
